# The determination of real fluid requirements in laparoscopic resection of pheochromocytoma using minimally invasive hemodynamic monitoring: a prospectively designed trial

**DOI:** 10.1007/s00464-019-06777-z

**Published:** 2019-04-11

**Authors:** Martin B. Niederle, Edith Fleischmann, Barbara Kabon, Bruno Niederle

**Affiliations:** 1 Department of Anesthesia, General Intensive Care and Pain Management, Medical University of Vienna, Waehringer Guertel 18-20, 1090 Vienna, Austria; 2 Division of General Surgery, Section Endocrine Surgery, Department of Surgery, Medical University of Vienna, Vienna, Austria

**Keywords:** Pheochromocytoma, Fluid management, Advanced intra-operative monitoring, Hemodynamics, Adrenalectomy, Preoperative preparation

## Abstract

**Background:**

Hemodynamic instability is frequently observed during adrenalectomy for pheochromocytoma (PCC). Guidelines recommend liberal preoperative volume administration. However, it is unclear whether fluid deficiency or vasoplegia causes shifting hemodynamics and whether minimally invasive hemodynamic monitoring with esophageal Doppler (EDM) can help visualize intraoperative changes avoiding volume overload and complications.

**Methods:**

Ten patients with biochemically verified PCC and five patients with hormonally inactive adrenal tumors (HIAT; control group) were treated following a strict protocol. During laparoscopic adrenalectomy, goal-directed fluid therapy was performed using EDM. Hemodynamic and biochemical data were documented. The primary outcome variables were fluid requirement and hemodynamic parameters.

**Results:**

Applying EDM, total intraoperative fluid administration was slightly higher in PCC patients than in patients with HIAT (2100 ± 516 vs. 1550 ± 622 ml, *p *= 0.097; 12.9 ± 4.8 vs. 8.3 ± 0.7 ml kg^−1^ h^−1^, *p *= 0.014). Hemodynamics varied considerably within the PCC group and was associated with type and level of secreted catecholamines. Arterial blood pressure and systemic vascular resistance index reached their minimum in the 10-min period after resection of PCC. Without liberal fluid administration, an increase in cardiac index was observed in both groups comparing baseline measurements to end of surgery. This increase was statistically significant only in PCC patients (PCC: 2.31 vs. 3.15 l min^−1^ m^−2^, *p *= 0.005; HIAT: 2.08 vs. 2.56 l min^−1^ m^−2^, *p *= 0.225).

**Conclusions:**

As vasoplegia, but not hypovolemia, was documented after tumor resection, there is no evidence that PCC patients profit from liberal fluid administration during laparoscopic adrenalectomy. To avoid volume overload, noninvasive techniques such as EDM should be routinely used to visualize the variable intraoperative course.

Trial registration: ClinicalTrials.gov, Identifier: NCT01425710.

Pheochromocytomas (PCC) are rare adrenal or extraadrenal catecholamine-producing tumors with an estimated incidence of approximately 0.46 to 4.65 per million inhabitants [1, 2]. Surgical resection is the only curative therapy to liberate the patient from its diverse symptoms [3, 4].

The surgical technique of choice is endoscopic adrenalectomy either through transperitoneal or retroperitoneal route [3] and is associated with low surgical morbidity and mortality [5–7]. However, the perioperative course is often complicated by hemodynamic instability, such as sudden hypertensive or tachycardic episodes during the initiation of pneumoperitoneum/pneumoretroperitoneum and tumor manipulation [8, 9], or severe and prolonged hypotension after tumor removal [7, 10].

Although various retrospective analyses have attempted to identify preoperative risk factors for intra- and postoperative complications, the perioperative course during tumor resection remains unpredictable [5, 7, 11, 12]. Current guidelines still recommend preoperative alpha blockade to avoid hypertensive events during resection and additionally a high-salt diet, liberal fluid intake and infusion therapy (approx. 2 l of saline the evening before surgery) to prevent postoperative hypotensive episodes [3, 13, 14].

Vasoplegia due to sudden fall of catecholamines, but not hypovolemia, has been suspected to be the reason for a substantial decline in arterial blood pressure (ABP) after tumor removal. Therefore, the usefulness of liberal perioperative fluid administration to prevent postresection hypotension has been questioned [8, 15, 16]. Additionally, an ongoing debate surrounds the potential danger and relation to postresection hypotension of preoperative alpha blockade [17–21]. To our knowledge, intraoperative fluid requirement has not been prospectively investigated as yet, although the potentially negative effects of liberal fluid administration are well recognized [22] and volume overload is present in at least 8% of patients after PCC resection [7].

To early recognize hemodynamic instability intraoperatively and to avoid complications postoperatively, advanced intraoperative hemodynamic monitoring has been recommended in recent guidelines [3, 14]. Initially, pulmonary artery catheter (PAC) [8, 20, 23–25], an invasive technique with potentially severe complications, was used. However, PAC contrasts the intention of endoscopic surgical procedures to minimize trauma and complications. Therefore, the use of noninvasive methods, such as esophageal Doppler monitoring (EDM) for visualizing cardiac output (CO), systemic vascular resistance (SVR) and fluid responsiveness, seems more rational.

EDM has been shown to be an effective method for advanced hemodynamic monitoring and goal-directed fluid therapy in various types of surgery [26–29].

Reviewing the literature, only two trials have been published using minimally invasive monitoring techniques during PCC surgery, both during open resection [15, 30]. To our knowledge, noninvasive monitoring systems have so far not been studied in endoscopic PCC removal. Therefore, this is the first study to apply EDM during laparoscopic adrenalectomy for PCC following a precise treatment protocol in an attempt to assess intraoperative fluid requirement and to visualize hemodynamic changes to elucidate the reasons for instability and to provide individualized treatment. Patients with PCC were compared with patients operated for hormonally inactive adrenal tumors (HIAT) receiving exactly the same surgical procedure.

## Materials and methods

The trial was conducted at the Departments of Anesthesia and Surgery, Medical University of Vienna. It was approved by the local ethics committee (EK 495/2011) and registered at ClinicalTrials.gov (Identifier: NCT01425710). All patients signed a written informed consent to all diagnostic and therapeutic procedures. All procedures were carried out in accordance with the Declaration of Helsinki and its later amendments.

### Patients

A total of 15 patients (PCC: *n *= 10, HIAT: *n *= 5 [controls]) were included in this prospective observational study. The patients’ baseline data are presented in Table 1.

**Table 1:** Baseline characteristics

Group	PCC	HIAT	*p* value
Patients (f:m)	10 (3:7)	5 (3:2)	–
Age (years)	56 (± 9)	62 (± 11)	0.253
Weight (kg)	78 (± 18)	82 (± 8)	0.663
Height (cm)	169 (± 7)	166 (± 10)	0.472
BMI (kg/m^2^)	27 (± 6)	30 (± 5)	0.354
ASA score	ASA 2: 9	ASA 1: 1	–
	ASA 3: 1	ASA 2: 4	
PCC-specific preoperative symptoms	HA: 4	–	–
	HTC: 2		
	TC: 1		
	None: 3		
Diabetes mellitus	3/10	0/5	–
Mean phenoxybenzamine end dose/day (mg)	25 (± 15)	0	–
Beta blockers	3/10	2/5	–
Other AHM	7/10	2/5	–
Max. tumor diameter (mm)	55 (± 30)	67 (± 38)	0.517
Duration of surgery (min)	139 (± 41)	136 (± 47)	0.904

Values are mean (± standard deviation) or number

*AHM* antihypertensive medication, *ASA* American Society of Anesthesiologists, *BMI* body mass index, *HIAT* hormonally inactive adrenal tumor, *PCC* pheochromocytoma, *TC* tachycardia

The histological evaluation revealed benign PCC (eight right-side, two left-side) and various benign adrenocortical lesions in the controls (two right-side, three left-side). Preoperative diagnosis of PCC was confirmed both biochemically (plasma nor-/metanephrines, urinary catecholamines and nor-/metanephrines in 24-h urine collection) and radiologically (fluorodopa positron emission tomography/computed tomography). Conn’s and Cushing’s syndromes were excluded biochemically in the HIAT group.

### Preoperative medical preparation

PCC patients received phenoxybenzamine (a nonselective, irreversible alpha blocker) starting 3 weeks before surgery. The initial dose was 5 mg daily for 1 week, then 10 mg in two doses daily for another week and, in the last week, increased by 10 mg every day as needed to achieve the target criteria recommended [31]. The patients were not given alpha blockers on the day of surgery. Already established beta blocker therapy was left unchanged. Other antihypertensive medication was reduced or discontinued to avoid hypotension. The patients did not receive any infusion therapy or special diet. In all subjects, concomitant structural heart disease was initially excluded by transthoracic echocardiography.

### Surgical procedure

In all patients, endoscopic adrenalectomy was performed with the transperitoneal flank approach. All tumors were removed en bloc with the surrounding fatty tissue. No selective lymphadenectomy was performed. The specimens were extracted in toto in plastic bags to avoid tumor spillage. Tumor size was measured directly after removal by the surgeon.

### Anesthesiological management

All patients were managed by one single anesthetist. Anesthesia was inducted via peripheral venous lines with continuous infusions of remifentanil at 0.5 µg kg^−1^ min^−1^ for 1 min followed by injections of 3 mg/kg propofol i.v. as bolus. To avoid any sympathetic activation by painful stimuli, a bispectral index of 40–50 was targeted and anesthesia was continued using sevoflurane (target minimum alveolar concentration 1.1) and continuous infusion of remifentanil at 0.3–0.4 kg^−1^ min^−1^. Pressure-controlled ventilation was used targeting a tidal volume of 6–8 ml kg^−1^ with a positive end-expiratory pressure of 5 mbar before and after pneumoperitoneum and 8 mbar during pneumoperitoneum at a respiratory rate of 10–16 min^−1^ to maintain normocapnia.

### Hemodynamic monitoring and fluid therapy

Before induction of anesthesia, all patients received an arterial line in the radial artery in order to invasively measure ABP. After intubation, a central venous catheter was placed into the right internal jugular vein. The EDM (CardioQ^®^, Deltex Medical Ltd., UK) was then installed and optimized as recommended by the manufacturer. Stroke volume (SV) and aortic corrected flow time (FTc) were recorded as proposed by the manufacturer and published previously [26]. Goal-directed fluid therapy was performed using balanced crystalloid fluid (Elo-Mel isotone^®^, Fresenius Kabi, Germany). Beside an initial bolus of 500 ml at the very beginning of surgery, boli of 250 ml was administered to target an FTc of 0.33–0.36 ms and continued if an at least 10% increase in SV was observed. A reassessment was done at least every 10 min or more often in the presence of hemodynamic instability. In the case of hypotension (mean ABP < 60 mmHg) and no response to fluid therapy, norepinephrine was initiated as continuous infusion. In the case of hypertension (systolic ABP > 180 mmHg or mean ABP > 100 mmHg), nitroprusside was administered continuously.

### Data collection

FTc, SV, CO and peak velocity (PV) data were collected continuously, and hemodynamic, respiratory and additional parameters were recorded at least every 2 min. The important time-points (incision, creation of pneumoperitoneum, ligature of suprarenal vein, tumor extirpation and end of surgery) were documented. The data were then summarized (mean) for the different intraoperative periods: period 1: intubation until incision; period 2: incision until creation of pneumoperitoneum; period 3: after creation of pneumoperitoneum until ligature of suprarenal vein; period 4: after ligature of suprarenal vein until tumor extirpation; period 5: first 10 min after tumor extirpation; period 6: after tumor extirpation until end of operation. Cardiac index (CI), stroke volume index (SVI), and SVR index (SVRI) were finally calculated using the formula by Mosteller to estimate body surface. Additionally, serum catecholamine levels were measured intraoperatively at seven predefined time-points.

### Postoperative outcome

All patients were brought to the post-anesthesia/intensive care units (PACU/ICU) directly after surgery. Hemodynamic parameters and adverse events were documented for 24 h.

### Statistical methods

Normal distribution was assessed with the Shapiro–Wilk test and visual histogram inspection. Data are given as mean values (± standard deviation) or median (25th quartile–75th quartile). Following descriptive analysis, the t test was used to compare fluid therapy within the groups and the Wilcoxon signed-rank test was used to compare period 1 to period 6 within each group. *p* values < 0.05 were considered significant.

## Results

### Fluid management

Using EDM for goal-directed fluid therapy, the amount of infusion administered intraoperatively was 2100 ml (± 516) in the PCC group as compared to 1550 (± 622) in the HIAT group (*p *= 0.092). After adjusting for the duration of surgery and body weight, patients with PCC received 12.9 (± 4.8) ml kg^−1^ h^−1^ and those with HIAT 8.3 (± 0.7) (*p *= 0.014). There was a trend toward more fluid given after tumor resection in PCC (about 300 ml additionally) that did not reach the level of significance (see Table 2).

**Table 2:** Intraoperative fluid therapy and balance

	PCC	HIAT	*p* value
Duration of surgery (min)	139 (± 41)	136 (± 47)	0.904
Total fluid given (ml)	2100 (± 516)	1550 (± 622)	0.092
Total fluid given per kg and hour (ml kg^−1^ h^−1^)	12.9 (± 4.8)	8.3 (± 0.7)	0.014
Blood loss (ml)	52 (± 6)	50 (± 0)	0.500
Urine output (ml)	328 (± 182)	218 (± 88)	0.229
Fluid balance	1720 (± 370)	1282 (± 580)	0.097
Fluid before tumor extirpation (ml)	1640 (± 600)	1450 (± 620)	0.589
Fluid after tumor extirpation (ml)	440 (± 360)	100 (± 140)	0.068

Values are mean (± standard deviation)

*HIAT* hormonally inactive adrenal tumor, *PCC* pheochromocytoma

### Hemodynamic and biochemical measurements

**Fig. 1:**
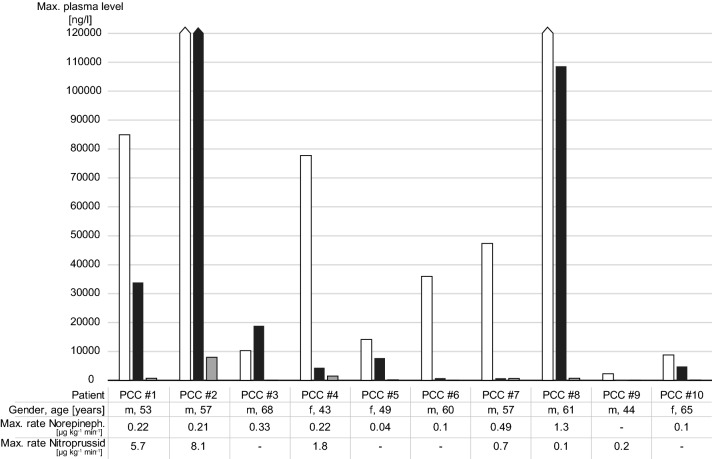
Type and maximum level of intraoperative catecholamine release [ng/l] and maximum intraoperative rate of vasoactive medication [µg kg^−1^ min^−1^] for each pheochromocytoma (PCC) patient. White bar: norepinephrine, black bar: epinephrine, gray bar: dopamine. Reference values: norepinephrine: < 420 ng/l, epinephrine: < 84 ng/l, dopamine: < 85 ng/l

**Fig. 2:**
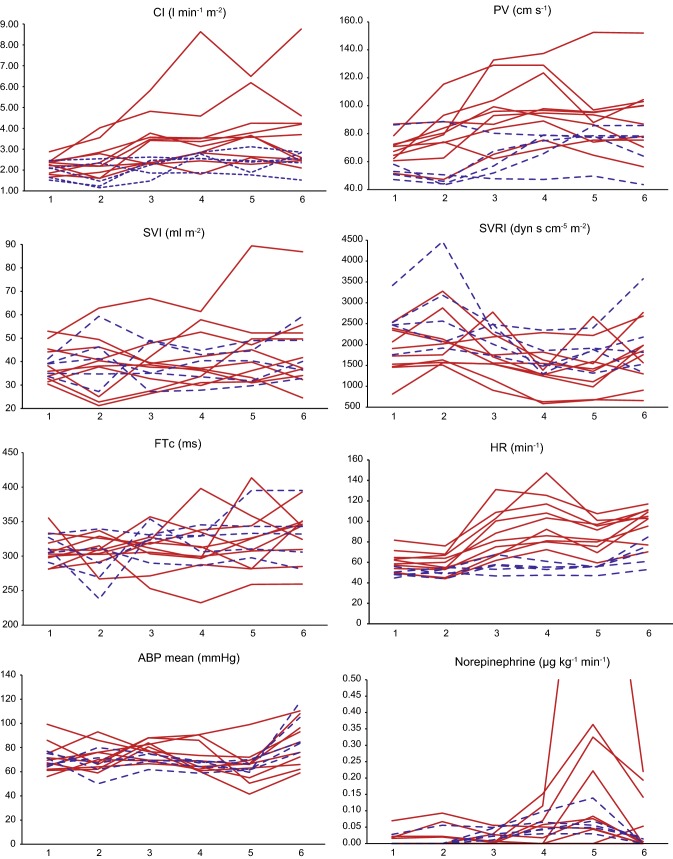
Intraoperative hemodynamic parameters. Solid red lines: patients with pheochromocytoma; broken blue lines: patients with hormonally inactive adrenal tumors. 1: Period 1—intubation until incision. 2: Period 2—incision until creation of pneumoperitoneum. 3: Period 3—creation of pneumoperitoneum until ligature of suprarenal vein. 4: Period 4—ligature of suprarenal vein until tumor extirpation. 5: Period 5—first 10 min after tumor extirpation. 6: Period 6—tumor extirpation until end of operation. *ABP* arterial blood pressure, *CI* Cardiac Index, *FTc* aortic corrected flow time, *HR* heart rate, *PV* peak velocity, *SVI* Stroke Volume Index, *SVRI* Systemic Vascular Resistance Index (Color figure online)

The type and maximum level of catecholamines released together with the maximum infusion rate of intraoperative norepinephrine and nitroprusside in PCC patients are presented in Fig. 1. Figure 2 summarizes the means of intraoperative hemodynamic parameters for the six predefined periods as individual curves for each patient.

The mean arterial blood pressure was held constant throughout surgery following the study protocol. Median FTc was within the targeted range at the end of surgery and increased in both groups.

All other hemodynamic measurements were very heterogeneous within the PCC group, whereas the patients with HIAT showed no marked differences (Fig. 2).

Especially in periods 3 (creation of pneumoperitoneum until ligature of suprarenal vein) and 4 (ligature of suprarenal vein until tumor extirpation), eight of ten (80%) patients with PCC showed a strong increase in CI during tumor manipulation. The four patients with the highest intraoperative peak CI (more than 4 l min^−1^ m^−2^) were among those with the highest intraoperative epinephrine blood levels (> 7000 ng/l; patients 3, 8, 5, 1). In accordance with CI, the PV peaked during tumor manipulation and then slowly decreased toward the end of surgery in all PCC patients but one (90%). Again, the three patients with maximum PV (above 120 cm/s) were among those with the highest epinephrine peak levels (patients 3, 5 and 8).

Intraoperative heart rate (HR) was constant in the HIAT patients and shifted markedly along the operations within the other group. Once more, there was a rise in HR in all patients with PCC (100%) during tumor manipulation (periods 3 and 4). The patients with the highest HR were again among those with markedly elevated epinephrine levels (patients 8, 2, 1, 3).

Table 3 presents the changes between baseline and end of operation. Both PV and HR increased significantly in the PCC patients (*p *= 0.007 and *p *= 0.005, respectively), while within the HIAT group, there was no difference in PV (*p *= 0.225) and a smaller but significant increase in HR (*p *= 0.043) comparing baseline measurements to end of surgery.

**Table 3:** Comparison between period 1 (before incision) and period 6 (end of surgery)

Period	PCC			HIAT		
	1	6	*p* value	1	6	*p* value
CI (l min^−1^ m^−2^)	2.31 (1.85–2.41)	3.15 (2.46–4.24)	0.005	2.08 (1.64–2.11)	2.56 (2.35–2.83)	0.225
SVRI (dyn s cm^−5^ m^−2^)	1815 (1473–2346)	1802 (1297–1982)^a^	0.646	2469 (2468–2528)	1813 (1528–2179)	0.345
PV (cm s^−1^)	69.1 (62.2–72.5)	93.4 (75.4–103.0)	0.007	53.0 (50.8–58.1)	77.4 (64.0–78.4)	0.225
SVI (ml/m^−2^)	37.5 (33.7–41.1)	37.0 (33.3–52.2)	0.139	38.2 (33.7–44.1)	49.7 (44.1–50.9)	0.500
FTc (ms)	304 (298–319)	344 (310–350)	0.06	311 (305–327)	332 (306–343)	0.273
MAP (mmHg)	68 (62–75)	80 (66–96)	0.203	71 (66–75)	84 (84–105)	0.043
HR (min^−1^)	60 (54–65)	104 (95–111)	0.005	48 (48–51)	71 (61–76)	0.043

Values are median (interquartile range)

^a^4 patients still on norepinephrine > 0.05 µg kg^−1^ min^−1^

*CI* Cardiac Index, *FTc* aortic corrected flow time, *HIAT* hormonally inactive adrenal tumor, *HR* heart rate, *MAP* mean arterial pressure, *PCC* pheochromocytoma, *PV* peak velocity, *SVI* Stroke Volume Index, *SVRI* Systemic Vascular Resistance Index

Table 4 focuses on the hemodynamic situation 10 min after tumor removal (period 5). In 8/10 (80%) patients with PCC, the lowest SVRI levels were reached within or immediately before this period (1/10 patients, 10%). Additionally, in this phase of surgery, the median SVRI of the patients in the PCC group was markedly decreased to 1337 (1244–1632) dyn s cm^−5^ m^−2^, although all PCC patients received their maximum norepinephrine dose within this period (see Fig. 1). Two patients showed a strongly decreased SVRI of lower than 1000 dyn s cm^−5^ m^−2^ (patients 3 and 8). Mean ABP was lowest in period 5 in all PCC patients but one (90%).

**Table 4:** Hemodynamic parameters (median, interquartile range) during period 5 (first 10 min after tumor removal)

	PCC	HIAT
CI (l min^−1^ m^−2^)	3.24 (2.83–3.54)	2.56 (2.54–2.77)
SVRI (dyn s cm^−5^ m^−2^)	1337 (1244–1632)	1528 (1482–1855)
PV (cm s^−1^)	90.8 (75.4–95.5)	78.4 (77.4–78.6)
SVI (ml/m^−2^)	34.7 (31.4–44.6)	50.0 (45.1–49.7)
FTc (ms)	317 (282–344)	333 (310–343)
MAP (mmHg)	66 (55–70)	62 (62–67)
HR (min^−1^)	87 (76–97)	56 (56–56)
Norepinephrine infusion rate (µg kg^−1^ min^−1^)	0.08 (0.04–0.33)	0.06 (0.05–0.07)

Values are median (interquartile range)

*CI* Cardiac Index, *FTc* aortic corrected flow time, *HIAT* hormonally inactive adrenal tumor, *HR* heart rate, *MAP* mean arterial pressure, *PCC* pheochromocytoma, *PV* peak velocity, *SVI* Stroke Volume Index, *SVRI* Systemic Vascular Resistance Index

### Postoperative outcomes

The postoperative course was uneventful in 12 patients. All but two patients with PCC left the operation room without catecholamine support. Of these two patients, one (patient 8) showed massively reduced SVRI (906 dyn s cm^−5^ m^−2^) but a sufficient CI of 4.6 l min^−1^ m^−2^ and FTc within the target range at 346 ms by the end of the surgical procedure. This patient required the highest intra-operative norepinephrine infusion. As the subject’s blood pressure could not be elevated to a mean arterial pressure (MAP) over 60 mmHg with norepinephrine alone, a vasopressin infusion was added (out of protocol). Both infusions were discontinued within 24 h after surgery. The second patient (patient 7) had a low SVRI of 1561 dyn s cm^−5^ m^−2^ but could be weaned from catecholamine within 6 h. Both patients received beta blockers prior to surgery (see Table 1) and had a HR of lower than the 25th percentile of the PCC group at the PACU/ICU.

Though experiencing normal MAP by the end of surgery, one other PCC patient required catecholamine support 2 h later. A hematoma was documented in the abdominal wall (max. diameter 9 cm) and blood pressure normalized after infusion of two units of red packed blood cells.

## Discussion

The perioperative management of PCC during endoscopic adrenalectomy requires close interdisciplinary cooperation between endocrine surgeons and anesthesiologists and has been under intensive debate [5, 14, 32].

Whilst guidelines recommend liberal fluid administration pre- and perioperatively to correct hypovolemia and to avoid hypotension after tumor resection [3, 14], some experts have challenged these recommendations [18, 33, 34]. Since intraoperative hemodynamics are extremely unpredictable, extended monitoring seems mandatory especially in large tumors to provide individualized therapy and to avoid overly liberal and potentially harmful fluid administration [15, 16, 18, 33], as found in at least 8% of PCC patients perioperatively [7].

To our knowledge, this is the first prospectively designed controlled trial to address hemodynamic changes and fluid demand during laparoscopic adrenalectomy in patients with PCC performing intraoperative noninvasive monitoring by using the esophageal Doppler technique.

The total amount of volume needed to optimize CO parameters was not excessively high in the PCC patients, even though these patients did not receive additional preoperative fluid therapy. The differences of 550 ml or 4 ml kg^−1^ h^−1^ between PCC and HIAT do not indicate that fluid deficiency was responsible for intra- or postoperative hemodynamic instability, since a substantial amount of the additional fluid was administered as “fluid trials” in response to low ABP after tumor resection. EDM visualized a reduction in SVRI directly after tumor removal in all patients with PCC. Additionally, prolonged hypotension was always associated with very low SVRI without signs of hypovolemia. These findings contrast guidelines suggesting fluid deficiency to be causative for hemodynamic instability [3, 14]. Our observations are in accordance with comparable previous studies using either pulmonary artery catheter [8] or performed during open surgery [15]. Vasoplegia was previously assumed to be responsible for hypotension [18]. However, prospectively collected data addressing the fluid status of PCC patients are still missing.

The marked increase in HR shown here was not observed in the only comparable study published by Joris et al. [8]. However, all patients in their investigation received beta blockers either intraoperatively or preoperatively. It is well known that hypotension due to SVR reduction (e.g., due to spinal anesthesia) results in both compensatory tachycardia and an increase in CO and is thereby best treated by applying alpha adrenoreceptor agonists but not excessive volume administration [35, 36]. Compatible to this, the two patients who required catecholamine infusions by the end of surgery in this study had normal or high CI but markedly reduced SVRI and failed to respond to fluid trials. Interestingly, both subjects were on beta blockers preoperatively and had low HR compared to the other PCC patients. Another patient (PCC #3) had comparably low SVRI (< 1000 dyn s cm^−5^ m^−2^) posttumor resection. This patient did not receive beta blocker therapy and had the highest CI, a HR of 110 bpm and normal ABP by the end of surgery. Thus, it seems possible that in the two patients with prolonged hypotension, the beta blockers inhibited compensatory tachycardia which is needed for normalization of ABP. Long-acting beta blockers should therefore be used with caution prior to surgery.

It cannot be excluded that reduction in SVRI in PCC patients is at least partially influenced by preoperative alpha adrenoreceptor blockade, as shown by Groeben et al. [19]. Although no patient in the present study received an alpha blocker on the day of surgery, individual pharmacodynamics could be responsible for the duration and amount of the postoperative effect, as the duration of phenoxybenzamine action may vary and depends on the velocity of alpha receptor resynthetization [37]. Advanced low-invasive hemodynamic monitoring may also assist in identifying these patients during operation with prolonged reduction in SVR who might profit most from advanced postoperative care [38].

Our data suggest that there is no rationale for preoperative fluid therapy as recommended in guidelines or even excessive fluid administration (up to 6 l of saline 0.9% within 48 h preoperatively have been reported even recently [39]). Normalizing hypertension with antihypertensive drugs (in our trial, treatment was started at least 3 weeks before surgery) should help “normalize” fluid status, if at all altered. Therefore, there is no rationale for any additional infusion therapy before surgery, as it is well understood that unnecessary administration of crystalloid fluids (especially saline) may have detrimental effects as well [22, 40]. The results of this trial thereby again underline that targeted and individualized volume management—increasingly claimed [41] in general—is essential but even more important for patients with cardiovascular abnormalities (such as catecholamine excess).

The fact is to be kept in mind that intraoperative courses may prove totally uneventful or definitively turbulent in PCC patients.

Closely monitoring the extremely diverse hemodynamic course during PCC surgery using minimally invasive techniques may help optimize and standardize anesthesiology management [42] and could avoid unnecessary or even harmful liberal use of fluids.

Although this trial was designed as an exploratory study and the small sample size may thus be a limitation, its strengths are its prospective and standardized study design and that the controls received the same surgery with an identical anesthetic protocol performed by the same anesthetist, thereby minimizing confounders. Therefore, providing a higher level of evidence than previous retrospective trials, our results may induce a critical reflexion of current perioperative concepts in perioperative management for laparoscopic adrenalectomy in PCC. Based on our preliminary data, a prospective randomized trial could compare different fluid management strategies regarding hemodynamic stability and outcomes in a larger number of PCC patients.

In conclusion, there is no evidence that PCC patients profit from liberal perioperative fluid administration. Reduction of SVRI, but not hypovolemia, is most likely the reason for hypotension after removal of a PCC. As hemodynamics and catecholamine levels vary extremely among PCC and are hardly predictable, EDM is a helpful and less invasive tool for advanced intraoperative monitoring and should be routinely used to provide individualized management during laparoscopic adrenalectomy for PCC.
